# The impact financial resources on implementation of large-scale electronic health records in the Saudi Arabia's primary healthcare centers: Mixed methods

**DOI:** 10.3389/fpubh.2022.1037675

**Published:** 2022-12-12

**Authors:** Haitham Alzghaibi, Yasir Hayat Mughal, Mohammad Alkhamees, Ibrahim Alasqah, Adel Sulaiman Alhlayl, Mohammed Hamed Alwheeb, Majedah Alrehiely

**Affiliations:** ^1^Department of Health Informatics, College of Public Health and Health Informatics, Qassim University, Albukayriyah, Saudi Arabia; ^2^Department of Health Administration, College of Public Health and Health Informatics, Qassim University, Albukayriyah, Saudi Arabia; ^3^Department of Public Health, College of Public Health and Health Informatics, Qassim University, Albukayriyah, Saudi Arabia; ^4^Department of Academic Directorate for Training and Research Affairs, Hail Health Cluster, Hail, Saudi Arabia; ^5^Department of Business Development, Institute of Research and Consulting Services, Prince Sattam University, Riyadh, Saudi Arabia; ^6^Department of Computer Science and Informatics, Applied College, AlUla, Saudi Arabia

**Keywords:** electronic health records, primary healthcare centers, financial resources, Saudi Arabia, large-scale projects

## Abstract

**Introduction:**

There is worldwide demand for the implementation of electronic health systems and a transformation to electronic transactions in healthcare organizations. This move to e-health transformation stems from the perceived positive impact that e-health systems have in improving the quality of healthcare and, in turn, reducing expenses. Despite this, more than half of previous Electronic Health Record System (EHRS) implementation projects have failed due to several barriers and challenges such as cost.

**Aim of the study:**

To evaluate the impact of financial resources (FR) on the implementation of the EHRS in PHCs in SA.

**Methods:**

A mixed methods approach was implemented. SPSS and AMOS-SEM are used to test reliability and validity and hypotheses. Thirty-one (59%) out of 51 policy makers at the MoH filled and returned the questionnaire while 13 policymakers were interviewed using semi-structure interviews.

**Results:**

Results revealed that both measurement model and structural models met the threshold. All scales are found reliable and valid. Furthermore financial resources have positive impact on EHRS implementation. Findings from both studies show that financial resources have a very positive impact to facilitate large-scale EHRs implementation and overcome barriers that may lead to the failure of the project.

## Introduction

Since the 1960s, Information Communication Technology (ICT) has been responsible for the performance enhancement and improvement of healthcare services (1, 2). The implementation of Information Technology (IT) in the last few decades of the twentieth century has led to a revolution in the way work is carried out and the way in which information is categorized and documented. The speed and precision that the IT revolution brought about made the governments of developed countries (where this revolution originated) immediately adopt these advanced, fast and efficient systems (3). Moreover, the implementation of the Electronic Health Record System (EHRS) has become a priority for both developed and developing countries (3). However, Deutsch, Duftschmid (4), Greenhalgh, Potts (5), and Smith (6), Madore, Rosenberg (7), Lorenzi, Kouroubali (8), Kruse, Stein (9) have argued that EHRS implementation is very complicated due to the shortage of experience with its implementation and the associated issues. Although, the barriers to EHRS implementation have been described, many of them remain unresolved (10). Therefore, it has been suggested that further research and investigation is necessary to overcome these barriers (10). According to Keshavjee, Bosomworth (11), Greenhalgh, Stramer (12), Lorenzi, Smith (13), Pare, Sicotte (14), and Yehualashet, Seboka (15), Ketikidis, Dimitrovski (16), around fifty percent of EHRS implementation projects around the world have failed. Others have estimated that the proportion of unsuccessful IT projects in the healthcare setting could be as high as seventy percent (17). In addition, according to Gagnon, Desmartis (18), the implementation of EHRS in Primary Healthcare Centers (PHCs) remains a greater challenge than its implementation in secondary care, such as hospitals. Cost of EHRS implementation needs to be considered at an early stage (pre-implementation phase) (19), as part of a readiness assessment (20). According to the studies of Cresswell, Bates (21), and Whitacre and Williams (22), Kemper, Uren (23), such initial costs can be a hindrance to organizations considering an EHRS. Previous research has identified two different types of cost related to EHRS implementation: start-up cost (initial cost), such as that associated with the purchase of a new system (24, 25); and ongoing costs, such as maintenance, training, developing infrastructure and provision of technical support (21, 26). Low financial resources can become a barrier to successful implementation (26–28). Kruse, Kristof (28), Simon, Kaushal (29), identified cost as a major barrier to EHRS implementation, whereas Fritz, Tilahun (30), in their systematic review, argued that financial resources were found to be a minor factor influencing EHRS implementation.

## Methods

This study was carried out at the Saudi Ministry of Health (MoH), which is based in the Ministry headquarters in Riyadh, the capital city of the Kingdom of Saudi Arabia. The Saudi MoH manages and oversees healthcare organizations in Saudi Arabia. Based on the last statistical yearbook that was published by the Saudi MoH, in 2020, the total number of employees was 430,096 (31).

### Questionnaire

Quantitative data were collected using a structured, self-administered questionnaire composed of pre-defined items and response options (32–34). In order to determine the influence of CPM on EHRS implementation, nine questions in the survey asked about the respondent's perception of the influence of this type of management on EHRS implementation. Questionnaire items were selected based on what was deemed to be the most influential factors to EHRS implementation (28, 35–39). The survey questionnaire as validated and piloted prior to use in the study.

### Semi-structured interviews

Qualitative data were collected using semi-structured interviews (40). The type of questions used were open-ended, in order to allow the participant the flexibility to describe their views and opinions (40). Semi-structured interviews allow us to expand on the questions following unexpected or interesting responses (41). Semi-structured interviews can also gather a wider variety of detailed data (42). Therefore, the main aim of conducting semi-structured interviews was to gain a comprehensive understanding and explanation of the role of FR on the process of EHRS implementation in PHCs. This approach was previously termed sequential explanatory mixed-methods (40).

### Population and sampling

The study population comprises all project team members directly or indirectly involved in implementing a large-scale EHRS project in Saudi PHCs. These consisted, for example, of heads of relevant departments (IT and PHC departments), senior managers, IT engineers, and technicians. This potential population of participants within the Saudi MoH has varying backgrounds and experience, departments, occupations and genders. The target sample was therefore all project team members (*n* = 53).

To reach the most appropriate subjects for this study (taking into consideration their involvement in the project implementation and knowledge they held about EHRS implementation in PHCs in SA), non-probability, purposive, snowball sampling was used (40, 43). For the qualitative purposes all project team members (*n* = 53), were invited to the semi-structure interviews, However, only 13 participated in the current study. The participants were occupied in five different positions General Manager (*n* = 3), Head of Department (*n* = 3), Deputy Head of Department (*n* = 1), Software Developer (*n* = 1), and Analyst (*n* = 5).

### Quantitative results

Out of the fifty-three, only thirty-one participated and completed the questionnaire, indicating a response rate of 59%. Reliability in this context is measured through a Cronbach's Alpha test which measures consistency in terms of percentages ranging from 0 to 100% (0–1). The study questionnaire was acceptable and had excellent reliability score (0.94).

Table 1 shows the percentage of male and female participation in the survey. It is evident that participation was male dominant, with 80.6% of participants being male. Female participation was found to be only 19.34%. This reflects the actual proportion of females and males in the Saudi MoH, where the majority of the staff are male, particularly the targeted population of this study. Further analysis revealed that the participants' role in the Saudi MoH, where the survey was conducted. The participants in the survey were found to be from diverse professional roles. The assistants formed the highest number of participants, at twenty-one (67.7%). Only one deputy manager participated in this study, and three participants from other positions. The table indicates that among the thirty-one participants, eighteen (58.1%) had been involved in previous EHRS implementation, and thirteen (41.9%) had never been involved in any EHRS implementation. shows the nature of the role played by the participants during EHRS implementation. This can be either direct involvement or cooperation with the process at various stages through indirect involvement. Out of thirty-one participants, twenty (64.5%) declared that they were directly involved in the process of implementation and five (16.1%) declared that they aided the process through an indirect connection. Six (19.4%) participants did not declare the nature of their involvement.

**Table 1:** Demographic information of respondents.

**Gender**	**N**	**Percent**
Male	25	80.6
Female	6	19.34
General manager	3	9.7
Deputy manager	1	3.2
Head of department	3	9.7
Deputy head of department	3	9.7
Assistant	21	67.7
Involvement in EHRS yes	18	58.1
No	13	41.9
Distribution role direct	20	64.5
Indirect	5	16.1

Participant responses to items representing the impact of financial resources on EHRS implementation in PHCs (Table 2).

**Table 2:** Participant responses to items representing the impact of financial resources on EHRS implementation in PHCs.

**Items**	**Strongly disagree**	**Disagree**	**Somewhat disagree**	**No opinion**	**Somewhat agree**	**Agree**	**Strongly agree**	**Median**	**Total agreement**	**Rank**
Overall impact is positive	–	–	–	1 (3.2%)	2 (6.5%)	3 (9.7%)	25 (80.6%)	7	30 (96.8%)	1
Better software selection	–	–	–	2 (6.5%)	2 (6.5%)	8 (25.8%)	19 (61.3%)	7	29 (93.5%)	2
Better team selection	–	–	–	2 (6.5%)	4 (12.9%)	14 (45.2%)	1,135.5%)	6	29 (93.5%)	3
Improve user training and motivation	–	1 (3.2%)	1 (3.2%)	1 (3.2%)	1 (3.2%)	5 (16.1%)	22 (71%)	7	28 (90.3%)	4
Improve provision of appropriate hardware	–	–	–	3 (9.7%)	1 (3.2%)	8 (25.8%)	19 (61.3%)	7	28 (90.3%)	5
Improve on-going support & maintenance	–	–	–	4 (12.9%)	2 (6.5%)	6 (19.4%)	19 (61.3%)	7	27 (87.1%)	6
Abundance of staff and professionals	–	1 (3.2%)	2 (6.5%)	1 (3.2%)	4 (12.9%)	4 (12.9%)	19 (61.3%)	7	27 (87.1%)	7
Appropriate infrastructure	–		2 (6.5%)	2 (6.5%)	1 (3.2%)	8 (25.8%)	18 (58.1%)	7	27 (87.1%)	8
Improve team communication	1 (3.2%)	1 (3.2%)	–	2 (6.5%)	3 (9.7%)	14 (45.2%)	10 (32.3%)	6	27 (87.1%)	9
Improve systems integration interoperability	1 (3.2%)		2 (6.5%)	2 (6.5%)	1 (3.2%)	9 (29%)	16 (51.6%)	7	26 (83.9%)	10
Improve organization structure redesign	1 (3.2%)	2 (6.5%)	1 (3.2%)	1 (3.2%)	2 (6.5%)	15 (48.4%)	9 (29%)	6	26	11

The impact was explored with eleven items (see Table 2). Overall, it was shown that there is a high agreement on all items (above 80%), some of which had slightly more agreement than others, with median scores being six or seven. It was shown that the influence of an abundance of financial resources is (1) “overall positive”, showing an agreement of 96.8% and a median score of seven, where participants strongly agree that an abundance of financial resources positively influences EHRS implementation. Whereas, the least agreement was given for Item (11) “Improve systems integration and interoperability” (83.9%).

Analysis of moment structure, structure equation modeling (AMOS-SEM) is used for development of structural and measurement model. Measurement model is developed to investigate the reliability and validity of the scales and items. Exploratory factor analysis (EFA) and confirmatory factory analysis (CFA) is used to cross check the reliability and validity of scales. According to Field (44) threshold for loadings in EFA must be >0.040, and for Cronbach Alpha >0.70 and according to Hair et al. (45) for CFA loadings must be >0.50 and for AVE >0.50 and CR >0.70, respectively. Results of EFA and CFA, Table 3 shows that all the values of loadings, AVE, CR and alpha met the threshold and thus author assumed that scales used in the current study for financial resources with 11 items and for electronic health record system, with eight construct 6 items for each construct are found reliable and valid. Thus now researcher can proceed toward development of structural model for testing hypotheses.

**Table 3:** Measurement model EFA & CFA (AMOS-SEM).

**Codes**	**Items**	**EFA loadings**	**CFA loadings**
**Financial resources**			
FR1	Better software selection	0.876	0.58
FR2	Better team selection	0.831	0.70
FR3	Improve the team communication.	0.820	0.97
FR4	Improve organization's workflow and structure redesign	0.686	0.91
FR5	Appropriate infrastructure.	0.726	0.76
FR6	Improve the on-going support and maintenance.	0.752	0.78
FR7	Improve the provision of appropriate hardware.	0.506	0.56
FR8	Improve users training and motivation.	0.752	0.80
FR9	Abundance in staff and professionals.	0.758	0.69
FR10	Improve systems integration and interoperability.	0.755	0.87
FR11	Overall impact is positive.	0.839	0.91
	**AVE (Average Variance Extracted) >0.50**	**0.566**	**0.597**
	**CR (Composite Reliability) >0.070**	**0.928**	**0.935**
	**A (Cronbach Alpha) >0.70**	**0.940**	
**Organizational readiness to large scale electronic health record**			
**system (EHRS)**			
1	Resources (6 items)	0.921	0.54
2	End user (6 items)	0.764	0.85
3	Technology (6 items)	0.799	0.85
4	Knowledge (6 items)	0.617	0.60
5	Process (6 items)	0.859	0.81
6	Values goals (6 items)	0.726	0.79
7	Management structure (6 items)	0.898	0.94
8	Admin support (6 items)	0.832	0.91
	**AVE (Average variance extracted) >0.50**	**0.652**	**0.636**
	**CR (Composite reliability) >0.070**	**0.937**	**0.931**
	**A (Cronbach alpha) >0.70**	**0.924**	

Bold values represent the convergent validity and reliability of the questionnaire.

### Regression analysis (structural model)

H_1_: There is Positive Significant effect of financial resources on electronic health record system implementation.

**Figure 1:**
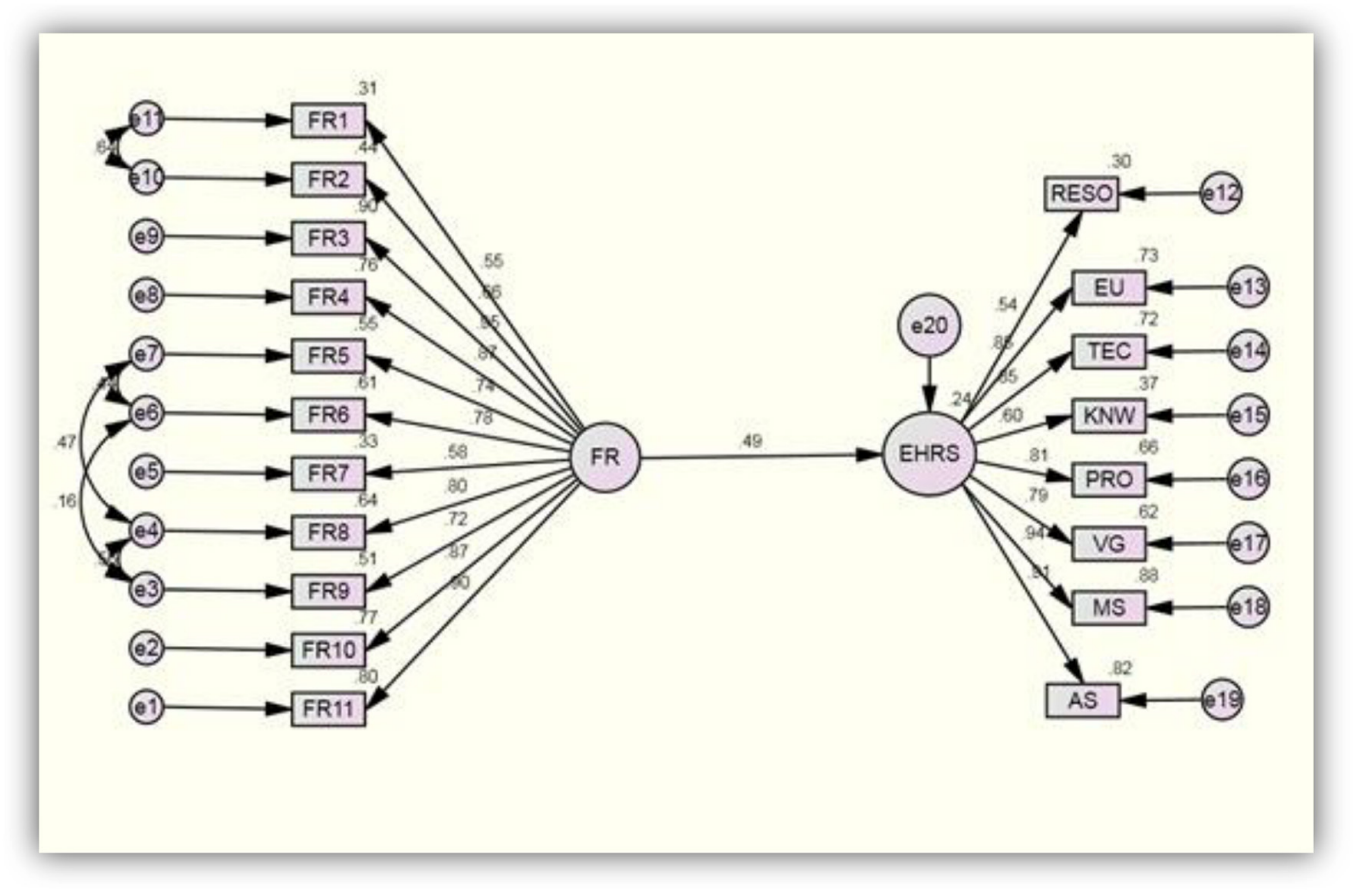
Structural model.

Structural model was developed in Figure 1 and results of regression analysis are presented in Table 4. Bootstrapping was run. From the above Table 4 it is evident that a financial resource has positive significant impact on electronic health record system (EHRS) i.e., β = 0.198, S.E = 0.090, CR (*t*-statistics > 1.96) = 2.201, *p* < 0.05. This implies that one percent increase in financial resources by Ministry of Health could possible increase implementation of EHRS all over primary healthcare centers in Saudi Arabia for 19.8%, respectively. Thus hypotheses 1 is accepted.

**Table 4:** Direct effects.

**Hypotheses 1**	**β**	**S.E.**	**C.R.**	***p***
FR → EHRS	0.198	0.090	2.201	0.028

Spearman's rho correlation coefficient was conducted between all nine scales to determine if there is any relationship between the impact of FR and the level of PHCs readiness to implement the EHRS. Spearman's rho correlation coefficient ranges between 0 and 1 (0–100%) and could be negative or positive. By looking at Table 5 it was evident that there is a significant positive correlation between participant's agreement with the impact of FR and all readiness scales (*p* < 0.05).

**Table 5:** Spearman's rho correlation coefficient between the 10 scales.

		**Readiness at resources level**	**Readiness at end-user level**	**Readiness at technological level**	**Readiness at knowledge level**	**Readiness at processes level**	**Readiness at values & goals level**	**Readiness at management structure level**	**Readiness at admin support level**	**Finance recourse impact**
Readiness at resources level	rho	1.000								
	*P*	.								
	*N*	31								
Readiness at end-user level	rho	0.672^**^	1.000							
	*p*	0.000	.							
	*N*	31	31							
Readiness at technological level	rho	0.717^**^	0.755^**^	1.000						
	*P*	0.000	0.000	.						
	*N*	31	31	31						
Readiness at knowledge level	rho	0.558^**^	0.705^**^	0.798^**^	1.000					
	*P*	0.001	0.000	0.000	.					
	*N*	31	31	31	31					
Readiness at processes level	rho	0.562^**^	0.672^**^	0.705^**^	0.808^**^	1.000				
	*P*	0.001	0.000	0.000	0.000	.				
	*N*	31	31	31	31	31				
Readiness at values & goals level	rho	0.586^**^	0.645^**^	0.591^**^	0.736^**^	0.816^**^	1.000			
	*P*	0.001	0.000	0.000	0.000	0.000	.			
	*N*	31	31	31	31	31	31			
Readiness at management structure level	rho	0.697^**^	0.640^**^	0.780^**^	0.717^**^	0.796^**^	0.728^**^	1.000		
	*P*	0.000	0.000	0.000	0.000	0.000	0.000	.		
	*N*	31	31	31	31	31	31	31		
Readiness at admin support level	rho	0.581^**^	0.650^**^	0.792^**^	0.808^**^	0.882^**^	0.720^**^	0.892^**^	1.000	
	*P*	0.001	0.000	0.000	0.000	0.000	0.000	0.000	.	
	*N*	31	31	31	31	31	31	31	31	
Finance recourse impact	rho	0.596^**^	0.415^*^	0.465^**^	0.280	0.479^**^	0.423^*^	0.517^**^	0.416^*^	1.000
	*P*	0.000	0.020	0.008	0.127	0.006	0.018	0.003	0.020	.
	*N*	31	31	31	31	31	31	31	31	31

* means significance level at *p* < 0.05 level,

** means significance at *p* < 0.01 level.

### Qualitative result

The participants were occupied in five different positions (see Table 6): General Manager (*n* = 3), Head of Department (*n* = 3), Deputy Head of Department (*n* = 1), Software Developer (*n* = 1), and Analyst (*n* = 3).

**Table 6:** Participant abbreviation description.

**Position**	**Code used**
General manager	GM
Head of department	HD
Deputy head of department	DHD
Software developer	SD
Analyst	Analyst

The Saudi MoH is characterized by an abundance of financial resources provided by the Saudi government, and the participants agreed upon this unanimously. Overall, FR has a very positive impact on EHRS implementation projects. All the participants reported that financial resources contributed positively and facilitated the success of many previous projects, in particular EHRS implementation projects, due to the country's ability to fund electronic transformation in all sectors and services. For instance, the participants said:

“*The role of FR is definitely positive; this country has more access to financial resources.”* (Analyst 3)

“*Positive, without a doubt.” (HD 1)*

“*FR is very positive.” (HD 3)*

“*The financial resources are the most important factor that contribute to the success of the project.”* (Analyst 1)

“*The main factor which helps us to implement EHRS is financial support.”* (SD 1)

Furthermore, a head of department stated that “*there are no financial problems that are hindering the implementation of the EHRS”* (HD1), and one of the general managers said “*the Kingdom does not suffer at all from the problem of availability of financial resources as they are available; plentifully and thankfully.”* (GM 1)

The provision of FR assists in the accomplishment of EHRS implementation projects in general. The Ministry had to fulfill all the decision-makers' requirements to complete the EHRS implementation project regardless of the cost, and was committed to providing them.

“*We have a high budget for the implementation of the EHRS both in hospitals and health centers.”* (GM 1)

“*Yes, we received an adequate budget for this project. It was supposed to provide a very strong balance sheet.”* (HD 3)

In regard to overcoming technical and other organizational challenges, the software developer revealed that a sufficient budget had been allocated for specific elements such as technical infrastructure and training.

“*We received major financial support to set up the infrastructure.” (SD 1)*

“*The Ministry appropriately financed the courses and provided housing and transportation for all trainees when they had to travel to attend courses at the Ministry.”* (SD 1)

Three percent of the Ministry's annual budget is allocated to the implementation of EHRS projects. This is stipulated in the policy of Saudi Arabia, where the same proportion of the budget of any ministry is allocated to information technology. In the case of the MoH, this amount is approximately three billion Saudi Arabian Riyals, which is equivalent to six hundred million pounds.

“*After developing the strategic plan of the Ministry, it was approved by the Council of Ministers who allocated three billion riyals (6 hundred million pounds) for adoption of IT in the MoH. It was the biggest budget ever for the Ministry to support IT implementation, and the support is still ongoing. The state policy has allocated 3% of the budget for any ministry for IT projects. This is very significant support and the figure was adopted annually, will cover all the costs of IT and will certainly facilitate the implementation of EHRS overall.”* (DHD 1)

### The impact of financial resources on the provision of hardware

All participants reported that the abundance of financial resources positively affects the availability of efficient, high-performance computers and other devices needed to run the EHRS without any issues.

“*Positive, one hundred percent.” (Analyst 1)*

“*…the availability of financial resources helps in providing high efficiency devices to ensure the system is running without any problems.”* (GM 1)

However, one of the analysts reported that the provision of hardware wasn't among the challenges of EHRS implementation in PHCs.

“*I don't think the provision of appropriate hardware is as big a problem as you think.” (Analyst 3)*

### The impact of financial resources on technical infrastructure

There was unanimous agreement that technical infrastructure is positively affected by an abundance of financial resources. A general manager stated that the abundance of financial resources has contributed greatly to overcoming all the challenges associated with development of the infrastructure, such as connectivity for new projects. Developing an appropriate infrastructure is very expensive, so abundant financial resources contribute to its facilitation.

“*FR helped us to overcome the obstacles we encountered with regard to infrastructure. Infrastructure is the toughest obstacle which we have encountered, but with money availability it became easy to overcome.”* (GM 1)

“*FR helps to provide the appropriate connection.”* (GM 2)

“*FR has a very positive impact, because preparing infrastructure is very expensive.”* (Analyst 1)

### The impact of financial resources on systems interoperability

One of the analysts argued that “*FR has no impact”* (Analyst 3). In addition, a software developer said, “*I do not think that financial support has an effect on EHRS interoperability”* (SD 1). However, the majority of participants confirmed that FR does have a positive impact on interoperability. FR assists in purchasing an effective standard, such as HL7. Moreover, FR allows organizations to select vendors that provide an interoperable EHRS.

“*FR helps in the purchase of a standard such as HL7.”* (GM 2)

“*The abundance of FR gives you the option of selecting appropriate vendors which can provide compatible systems and not be limited to less expensive companies whose systems may not be compatible with other EHRS.”* (DHD 1)

“*FR has a positive effect by providing high quality standards and making EHRS compatible with each other.”* (Analyst 1)

### The impact of financial resources on PHC restructuring and workflow redesign

The majority of respondents stated that FR has a positive influence on restructuring and workflow design. For instance, GM2 said “*FR helps to redesign the PHC workflow”*. The Saudi MoH was able to sign a contract with a specialist company to assist them during the restructuring of the PHCs to be ready for the new EHRS implementation.

“*FR helps to sign a contract with consulting firms specializing in business re-engineering.”* (DHD 1)

However, others believe that FR has limited and even no impact on PHC restructuring and workflow redesign.

“*Very limited impact on this matter, but it is a positive.”* (HD2)

“*This matter has nothing to do with FR, from my point of view.”* (Analyst 1)

### The impact of financial resources on software selection

All participants agreed that FR has a very positive impact on the selection of an appropriate and highly efficient EHRS. FR facilitate the selection of the most efficient system. For example:

“*Definitely has a positive impact.” (Analyst 3)*

“*Abundant FR are positive, as in such case the Ministry selects an excellent system.” (Analyst 1)*

“*We have the option to choose the best system available, so it is very positive.”* (DHD 1)

Furthermore, software selection is highly influenced by FR. Thus, the MoH is not restricted to certain systems due to a lack of financial resources.

“*Software selection is highly affected by FR, where it gave us a big chance to choose any EHRS without being confined to specific systems due to shortage in FR.”* (HD 3)

### The impact of financial resources on project team communication

The majority of the statements were in agreement with the positive impact of FR on project team communication. Project team communication is one of the costliest procedures, and FR contribute to overcoming this challenge, especially when the Saudi MoH holds meetings between members from different regions.

“*Joint collaborative work between project team members in different regions is very expensive, and the money was available and helped us a lot.”* (GM 1)

“*FR has a positive effect here, it was easy for us to hold meetings with all members from different regions, we also arranged visits to the PHCs for consultation and evaluation. FR were always available for these activities.”* (HD 3)

### The impact of financial resources on project team selection

The participants agreed that there are professional shortages in HI and IT. However, FR assisted the Saudi MoH in attracting qualified personnel to participate in the EHRS implementation project. Thus, FR are considered to have a positive impact on team selection.

“*It is positive. The formation of an excellent project team, large amounts of money paid to hire experts.”* (HD 1)

“*The availability of FR assists in hiring talents and experts, otherwise we would have had big problems and select unqualified people, which may affect the implementation of such large-scale projects.”* (GM 1)

*The financial support has had a very positive impact on the provision of experts.” (*Analyst 2)

However, others argued that IT and HI professional's availability is a worldwide issue and FR have no impact on the provision of those individuals.

“*It is not a cost issue, it is a worldwide shortage, so I don't think FR has a role.”* (Analyst 3)

“*In terms of expertise and competencies, I do not think FR has an effect.”* (GM 3)

### The impact of financial resources on the provision of technical support

Once again, the participants unanimously agreed on the positive impact of an abundance of FR on the provision of technical support. The provision of technical support is very costly. Thus, with sufficient money, fifteen to twenty percent can be added to the cost of the contract fees for continuous technical support, maintenance and insurance. Thus, the Saudi MoH can ensure the success of the EHRS implementation project. Moreover, FR contribute positively by paying for technical support 24 h per day, with the MoH compensating the technicians for excessive working hours.

“*FR are positive because if we buy the EHRS we can add 18 or 20% to the project cost to provide continuous technical support as well as insurance and system maintenance.”* (GM 1)

“*It has a positive effect because technical support should be continuous, thus extra pay for additional work hours or more technicians is necessary, and this requires providing large amounts.”* (Analyst 1)

### The impact of financial resources on the provision of training

None of the participants reported that the abundance of FR has a negative or neutral impact on the training process. All agreed that an abundance of FR has a positive impact on the training process. For example, Analyst 3 said “*Absolutely, FR has a positive impact”* on provision of training, HD1 said, “*It has a very positive effect.”* Analyst 2 said, “*FR is essential and has a very positive impact, particularly on training.”*

## Discussion and conclusion

Overall, findings illustrated that FR had a very high positive impact on facilitating the implementation of large-scale EHRS in the PHCs and contributing to overcome many challenges. The findings showed that the Saudi MoH did not face any financial constraints during the implementation of the EHRS projects. Thus, the influence of this factor has been examined against some of the main factors found to have a direct relationship with FR. This study is the first comprehensive investigation of the impact of the FR on EHRS implementation, whereas the impact of FR has been examined against a wide range of factors which have been presented in previous literature. Consequently, the factor most influenced by FR was software selection, where 93.5% of project team agreed that FR assists in the selection of high-quality software. It was perceived that FR could have a beneficial effect on software selection, allowing more flexibility to select the best vendors to implement EHRS in PHCs and then enhance the system interoperability. Although, preparing adequate infrastructure is very costly (46), it was another factor that significantly influenced by the provision of the FR in a positive way. Another interesting finding from this study was that FR facilitated the provision of training and technical support which had previously been reported as a barrier to implementing a large-scale EHRS. Ninety percent of the participants agreed that FR has a very positive impact on the provision of training. In addition, in a semi-structured interview, Analyst 2 said, “*FR is essential and has a very positive impact, particularly on training”*.

Although findings of this study illustrated that FR was one of the main facilitators to the implementation of the EHRS in Saudi PHCs, others found that the cost of implementation was one of the main barriers, and the Saudi healthcare organizations struggle to support their project due to FR shortages (47–49). Likewise, internationally, the cost of EHRS implementation is classified as a barrier to the success of the projects [(24), e.g., (27, 28, 50–56)]. It is worth noting that my study is the only one which has examined the impact of the FR to on the implementation of large-scale EHRS in the PHCs.

## Data availability statement

The raw data supporting the conclusions of this article will be made available by the corresponding authors, without undue reservation.

## Ethics statement

This study is part of larger study named “Improve a strategy to implement the EHRS on widespread PHCs in developing countries with CMs organisations”. It was approved by the “Research Ethical committee at School of Health Science, Swansea University”. The research was conducted in accordance with: https://www.hra.nhs.uk/planning-and-improving-research/policies-standards-legislation/uk-policy-framework-health-social-care-research/ UK Policy Framework for Health and Social Care Research – Health Research Authority (hra.nhs.uk) and the Institutional Review Board (IRB) of the King Fahad Medical City (KFMC) at the Saudi MoH (IRB Log No. 14-189E). This research was in accordance with the declaration of KFMC and King Abdulaziz City for Science and technology (KACST) (H-01-R-012). In the case of questionnaire-based study, all participants were informed of the study. All participants provided written informed consent prior to enrolment in the study.

## Author contributions

HA: conceptualization, methodology, data collection, validation, analysis, and writing. YM: conceptualization and reviewing and editing. All authors contributed to the article and approved the submitted version.
